# Role of the aphid species and their feeding locations in parasitization behavior of *Aphelinus abdominalis*, a parasitoid of the lettuce aphid *Nasonovia ribisnigri*

**DOI:** 10.1371/journal.pone.0184080

**Published:** 2017-08-30

**Authors:** Govinda Shrestha, Henrik Skovgård, Gadi V. P. Reddy, Tove Steenberg, Annie Enkegaard

**Affiliations:** 1 Western Triangle Ag Research Center, Montana State University, Conrad, MT, United States of America; 2 Department of Agroecology, Science and Technology, Aarhus University, Research Centre Flakkebjerg, Forsøgsvej 1, Slagelse, Denmark; Institut Sophia Agrobiotech, FRANCE

## Abstract

Aphid species feeding on lettuce occupy distinct feeding sites: the lettuce aphid *Nasonovia ribisnigri* prefers to feed on heart leaves, whereas the potato aphid *Macrosiphum euphorbiae* feeds only on outer leaves. The aphid parasitoid *Aphelinus abdominalis*, known to be able to regulate *M*. *euphorbiae* on many crops, has recently been indicated as a promising biocontrol candidate also for use against *N*. *ribisnigri*, a major pest of lettuce. This study therefore examined *A*. *abdominalis* parasitization preference between *N*. *ribisnigri* and *M*. *euphorbiae* and its ability to parasitize aphids feeding on different parts of lettuce plants. In addition, life history traits of *A*. *abdominalis* on these aphid species were investigated. In no-choice laboratory experiments on leaf discs and 24 h exposure, *A*. *abdominalis* successfully parasitized 54% and 60% of the offered *N*. *ribisnigri* and *M*. *euphorbiae*, respectively, with no significant difference. In the corresponding choice experiment, however, *A*. *abdominalis* had a tendency for a significantly higher preference for *M*. *euphorbiae* (38%) compared to *N*. *ribisnigri* (30%). Growth chamber experiments on whole plants demonstrated that *A*. *abdominalis* was able to parasitize aphids, regardless of their feeding locations on lettuce plants. However, aphid feeding behavior had a significant effect on the parasitization rate. *A*. *abdominalis* parasitized significantly higher percentages of *M*. *euphorbiae* or *N*. *ribisnigri* when aphids were exposed separately to parasitoids on whole lettuce plants as compared with *N*. *ribisnigri* exposed only on heart leaf. A significant preference of *A*. *abdominalis* for *M*. *euphorbiae* compared to *N*. *ribisnigri* was also observed in the growth chamber choice experiment. A high percentage of adult emergence (> 84%) and female-biased sex ratio (> 83%) were found irrespective of the aphid species.

## Introduction

Infestation by aphids is a serious problem in the production of lettuce, *Lactuca sativa* L. (Asterales: Asteraceae), both in glasshouses and under field conditions [1]. Although several species of aphid occur on lettuce globally [2], the green peach aphid *Myzus persicae* (Sulzer), potato aphid *Macrosiphum euphorbiae* (Thomas) and lettuce aphid *Nasonovia ribisnigri* (Mosley) (Hemiptera: Aphididae) are of major importance [3, 4, 5]. Out of these, *N*. *ribisnigri* is the most critical due to its frequent occurrence throughout the growing season and its cryptic feeding habitat with a feeding preference for the heart leaves of lettuce [6, 7]. The other two species *M*. *persicae* and *M*. *euphorbiae* are usually considered of lesser economic importance as they only feed on outer lettuce leaves [6] and occur less frequently during the growing season [4, 5, 8].

Control strategies for *N*. *ribisnigri* populations on lettuce rely largely on the use of insecticides [9, 10, 11]. However, demand for alternative methods to control *N*. *ribisnigri* has been stimulated due to the increased risk of insecticide resistance in aphid populations [12, 13], and because of concerns related to the environment [14] and human health [15]. Potential biocontrol methods for *N*. *ribisnigri* comprise the use of predators, including syrphids [16, 17] and lacewings [18, 19], and fungal pathogens [20, 21]. A further potential method for *N*. *ribisnigri* biocontrol is the use of parasitoids [22].

Shrestha et al. [22] evaluated three commercially available parasitoid species for their potential against *N*. *ribisnigri* and found *Aphelinus abdominalis* (Dalman) (Hymenoptera: Aphelinidae) to be the most promising candidate. This parasitoid is believed to originate from Europe, but now occurs also in Asia and North America [23]. *A*. *abdominalis* has been used for biocontrol of *M*. *euphorbiae* in glasshouse and field crops [23, 24]. No information, however, is available regarding the parasitization preferences of *A*. *abdominalis* towards *N*. *ribisnigri* and *M*. *euphorbiae*, which appear simultaneously in lettuce fields. It is thus important to further evaluate the potential of *A*. *abdominalis* against *N*. *ribisnigri*, taking into account that feeding behavior may influence the degree to which an aphid species is parasitized [25, 26].

Parasitoids that attack more than one aphid species show differences in preference and performance in response to various aphid species [27, 28, 29, 30, 31, 32, 33, 34]. The preference behavior of parasitoids between aphid species or taxa is influenced by a number of factors such as 1) host quality, with better quality hosts species usually, but not always [31], being preferred over poor quality hosts [27, 28]; 2) color of aphid morph, with a greater preference for the green morphs compared to the red morphs [35, 36]; 3) aphid size, with smaller aphids usually, but not always [37, 38], being preferred over larger ones [27, 39]; and 4) aphid age, with a stronger preference for young or intermediate growth stages of aphids over old stages [40, 41]. Additional factors that influence parasitoid preference are: parasitoid age, with a greater preference for low quality hosts by short-lived than longer-lived parasitoids [42], and parasitoid egg load, with females with low egg load preferring high quality hosts compared with females with high egg loads [43].

Aphid parasitoid life-history traits such as offspring survival and offspring sex ratio are parameters commonly measured to evaluate parasitoid fitness on different host species [29, 31, 44]. Aphid parasitoids may be able to regulate the fitness of their offspring in relation to the host species they attack [27, 29, 31, 44]. Ovipositing females may allocate male and female offsprings differentially in different host species. Moreover, different host aphid species may produce changes to the survival of male and female offspring. [27, 29, 31, 44]. It is therefore important for biocontrol programs to investigate whether host aphid species influence parasitoid offspring sex ratio or survival. In addition, the capacity of parasitoids to locate hosts in their feeding sites is vital for the efficiency of a parasitoid as a biocontrol agent [25, 26]. Some parasitoids have the capability to find and parasitize aphids feeding on concealed parts of plants [45, 46] and vice versa [46].

None of the above-mentioned aspects have been explored in relation to use of *A*. *abdominalis* against *N*. *ribisnigri* co-occurring with *M*. *euphorbiae*. This study therefore examined the parasitization preference of *A*. *abdominalis* with regard to *N*. *ribisnigri* and *M*. *euphorbiae* under laboratory conditions and its capacity to find and parasitize the two aphid species when they are feeding on different areas of the lettuce plant under growth chamber conditions. In addition, female sex ratios and successful adult emergence of *A*. *abdominalis* on the two aphid species were also studied to evaluate parasitoid fitness.

## Materials and methods

### Plants

Iceberg lettuce, *L*. *sativa* cv. ‘Mirette’ was used as a source of plant material for the laboratory and the growth chamber experiments. Seeds were sown on Jiffy-strip trays and maintained in a glasshouse at 15–18 ^o^C, 55*–*70% RH and natural light conditions until three true leaves had emerged (approx. 2 weeks after seed sowing). Afterwards, plants were transplanted into 2 L pots filled with peat soil, perlite and vermiculite (mixed at 90:8:2) with a *pH* of 6–7. These plants were either utilized within 6–10 days for production of aphid cohorts, for rearing of parasitized vs. unparasitized aphids (detached leaflets, lab and growth chamber experiments) or maintained for additional three days in a glasshouse and subsequently transported to the growth chamber.

### Insects

The lettuce aphid *N*. *ribisnigri* and the potato aphid *M*. *euphorbiae*, originally supplied by Dr. Gemma Hough (Warwick Crop Centre, University of Warwick, UK) and senior research scientist Lesley Smart (Department of Biological Chemistry and Crop Protection, Rothamsted Research, UK), respectively, were reared separately on iceberg lettuce plants inside the insect- proof net-covered cages (68 × 75 × 82 cm). They were maintained in a controlled environment glasshouse compartment at 22 ± 1 ^o^C, 70 ± 5% RH and 16:8 L: D.

The parasitoid *A*. *abdominalis*, supplied as mummies by EWH BioProduction, Tappernøje, Denmark, were placed in plastic Petri dishes (diameter: 15 cm) and kept in a climate cabinet at 22 ^o^C, 70 ± 5% RH and 16:8 L:D. Mummies were checked daily for adult emergence and the cohorts of adults emerging on a same day were reared until the age of three days with the technique described by Shrestha et al. [22].

### Aphid cohorts

The cohorts of 2-3^rd^ instar aphids of *N*. *ribisnigri* and *M*. *euphorbiae* were used for laboratory and growth chamber experiments since these stages of both aphid species have been reported suitable for parasitization by *A*. *abdominalis* [47, 48]. To obtain cohorts of the two aphid species, adults (10–12 days old) were carefully transferred from the stock culture to uninfested leaves of lettuce. The base of each leaf was wrapped with moist cotton, inserted into a 1.5 ml Eppendorf tube with demineralized water and then placed at the bottom of mesh screened Plexiglass box (17 × 11 × 3 cm) with moist filter paper. These boxes were kept in a climate cabinet at 22 ^o^C, 70 ± 5% RH and 16:8 L: D. After 48 hours, the produced nymphs were gently transferred either to new clean leaves (Eppendorf tube and Plexiglass set up) for the laboratory experiments or to the clean plants for the growth chamber experiments. Aphids were maintained for additional two days for the nymphs to develop into 2-3^rd^ instars at similar conditions as described above [49, 50].

### Laboratory experiments: Parasitization preference and life history traits

#### No-choice tests

The no-choice experiments were performed to evaluate the parasitization rates and fitness of *A*. *abdominalis* on *N*. *ribisnigri* and *M euphorbiae* by measuring parasitism events (both successful and incomplete, i.e. without mummy formation) as well as a parasitoid emergence rates and sex ratios. The experiment was performed in vented Petri dishes (diameter: 9 cm) lined with a moist filter paper. A circular lettuce leaf disc (diameter: 5 cm) was placed at the bottom of each dish. Twenty aphid individuals of 2-3^rd^ instar, either of *N*. *ribisnigri* or *M euphorbiae*, were transferred to each lettuce dish by using a fine camel hair brush. Aphids were allowed to settle on a leaf disc for one hour before the introduction of a female parasitoid.

One mated female parasitoid (4 days old) was released into each Petri dish arena containing *N*. *ribisnigri* or *M euphorbiae* and left for a 24 hour parasitization period in a climate cabinet at 22 ^o^C, 70 ± 5% RH and 16:8 L: D. The female parasitoid was subsequently removed and the number of dead and live aphids in each dish counted under a stereo microscope. The numbers of aphids dying due to host feeding by *A*. *abdominalis* was determined based on their shrunken appearance [51]. The live aphids of each leaf disc were transferred to two clean leaves with the petiole wrapped with moist cotton and inserted into a 1.5 ml Eppendorf tube with demineralized water. This was done to avoid degradation of the leaves. These two leaves were placed in a Plexiglass box with moist filter paper and incubated in a climate cabinet under similar conditions as described earlier. After 4–5 days, the filter paper was replaced and if necessary, a new fresh leaf was placed in the vicinity of the old leaf to allow the aphids to translocate themselves. Generally, lettuce leaves remained fresh for at least six days using this setup.

Aphids were checked at 1–2 day intervals for two weeks for appearance of mummies (successful parasitization), while only up to nine days for aphids that died without mummification (incomplete parasitization). Aphid mummies that formed in each dish were gently collected using a fine camel hair brush and transferred individually into small transparent medicine cups (diameter = 15 mm) with screened lids. Emergence of adult parasitoids was checked at 1–2 day intervals and emerged parasitoids sexed under a stereo microscope. For each treatment, 12–14 replicates were performed. For the controls, five replicates without addition of parasitoids were used for each aphid species and same procedure as above was followed.

#### Choice test

The choice experiment was conducted in order to assess the preference of *A*. *abdominalis* for parasitization with regard to *N*. *ribisnigri* and *M*. *euphorbiae*. The experimental procedures and experimental conditions were similar as described above except that cohorts of 2-3^rd^ instar lettuce aphids and potato aphids (n = 20+20) were offered simultaneously on the same leaf disc. The lettuce aphid nymphs were introduced first and allowed to settle for 15 min prior to the releases of the potato aphid nymphs. *N*. *ribisnigri* nymphs are easily distinguished under a stereo microscope by their color (red) in contrast with whitish-green potato aphids. The number of replicates for treatment was 15 and the controls (replicates = 5) were performed without addition of parasitoids.

### Growth chamber experiments: Aphid feeding locations

The growth chamber experiments were performed to assess whether the aphid feeding location preference on lettuce plants influences the host finding ability of *A*. *abdominalis* by measuring successful parasitization under no-choice and choice conditions. Lettuce plants established in the plant growth chamber were 28 days old after seeding and had five unfolded leaves (4 outer leaves and 1 heart leaf) at the time of experiment initiation. A leaf developed from the central portion of plants was denoted as heart leaf and the leaves developed from peripheral layers as outer leaves. Plants were drip irrigated daily for half an hour each morning and evening in the growth chamber room and they were maintained at 22 ^o^C, 70 ± 5% RH and 16:8 L: D, for the duration of the experimental period.

#### No-choice and choice tests

The no-choice tests consisted of three treatments: 1) *M*. *euphorbiae* inoculated on leaves of a lettuce plant, 2) *N*. *ribisnigri* inoculated on leaves of a lettuce plant and 3) *N*. *ribisnigri* inoculated on only the heart leaf of a lettuce plant. Fifty 1^st^ instar aphids were inoculated on each plant in all three treatments (see section aphid cohorts), but the number of aphid individuals inoculated into each leaf of a lettuce plant varied among the treatments. In treatment 1, *M*. *euphorbiae* individuals were inoculated on outer leaves (4 leaves at the time of inoculation) with 12–13 individuals (totaling 50 aphid individuals) per leaf since it known that this aphid species does not colonize the heart leaves [6]. In treatment 2, *N*. *ribisnigri* individuals were inoculated on all five leaves (4 outer leaves and 1 inner leaf) with 10 individuals per leaf because this aphid species is known to colonize not only to heart leaf but also on outer leaves [7]. In treatment 3, 50 *N*. *ribisnigri* individuals were inoculated only on the heart leaf and the outer leaves were removed one day before the aphids’ introduction, as the heart leaf is the most preferred feeding site of *N*. *ribisnigri* on lettuce plant [7]. The removal of outer leaves in treatment 3 was done to avoid the movement of aphids to outer leaves and also to obtain the best estimate of *A*. *abdominalis’* parasitization on aphids situated on this leaf.

With respect to the choice test, fifty 1^st^ instar nymphs of each aphid species (totaling 100 aphid individuals) were established simultaneously on each lettuce plant. The inoculation of *M*. *euphorbiae* or *N*. *ribisnigri* individuals was carried out in a similar fashion is as in treatment 1 and 2 in the no-choice tests, respectively.

From this point forward, the experimental procedures for both choice and no-choice tests were the same. Plants established in the growth chamber were transported to the insect inoculation chamber, where the aphids were carefully inoculated on the dorsal side of the leaves by using a fine camel hair brush. Aphids were allowed to settle on the plants for 1–2 hours after which the plants were subsequently transported back into their original location in the growth chamber. Each aphid-inoculated plant was kept separately in an acrylic cylindrical insect cage (diameter = 18 cm and height = 12 cm) with 5–6 mesh screened holes (diameter = 5 cm) on the side. Forty-eight hours after inoculation, when aphids were allowed to distribute themselves on the plants and develop into 2–3 instar aphids, five female *A*. *abdominalis* parasitoids (mated, 4 days old) were released onto the top of the plant canopy of each aphid-inoculated plant. Parasitoids were then allowed to parasitize for 48 hours. Afterwards plants were transported to the insect inoculation chamber and carefully removed from pots in order to minimize the loss or escape of aphids.

The number of live aphids present on each plant leaf was counted and each aphid transferred onto uninfested leaves (Eppendorf tube and Plexiglass set up) and incubated in a climate cabinet at 22 ^o^C, 70 ± 5% RH and 16:8 L: D for 2 weeks. The subsequent handling of the aphids as well as the scoring of data was conducted as described above for the laboratory experiment. There were twelve replicates (each plant = one replicate) for each treatment for the both no-choice and choice tests. The controls (replicates = 5) were performed in absence of any parasitoids.

### Statistical analysis

The data were analysed in R 2.15.1 [52]. For all data, a normal quantile-quantile plot was first performed to check the normality of residuals and the equality of residual variances. A transformation (angular) was done to achieve normal distribution prior to statistical tests. Tukey contrast pairwise multiple comparisons were used to test for significant differences in means [53].

For the laboratory data set (Petri dish setup), one way analysis of variance (ANOVA) was performed to test the effect of aphid species on the percentage of successful parasitization and incomplete parasitization in the no-choice experiments and for any differences in successful parasitization or incomplete parasitization when two aphid species were offered simultaneously in the choice experiment. The percentage of successful parasitism was calculated as (Number of mummified aphids/Total numbers of aphids exposed minus host feed aphids) × 100 and incomplete parasitism as (Number of corrected dead aphids without signs of mummification/Total numbers of aphids exposed minus host feed aphids) × 100 [22]. Dead aphids recorded in the incomplete parasitization group [54] were corrected for control mortality [55] prior to calculation and statistical analysis.

Similarly, for the growth chamber data set, one way analysis of variance (ANOVA) was performed to examine the effect of aphid species feeding sites on successful parasitization percentage in the no-choice experiment and for any differences in successful parasitization when two aphid species were offered simultaneously in the choice experiment. The percentage of successful parasitism was calculated as (Number of mummified aphids recorded per plant/Total numbers of aphids exposed) × 100.

The adult emergence and sex ratio data were found to be non-normally distributed even after the angular transformation and the non-parametric one-way analysis of variance, Kruskal-Wallis test, was therefore used to test for differences. A Mann-Whitney U-test was used as a post hoc test for multiple comparisons between the means.

## Results

### Laboratory experiment

#### Parasitization

**Fig 1:**
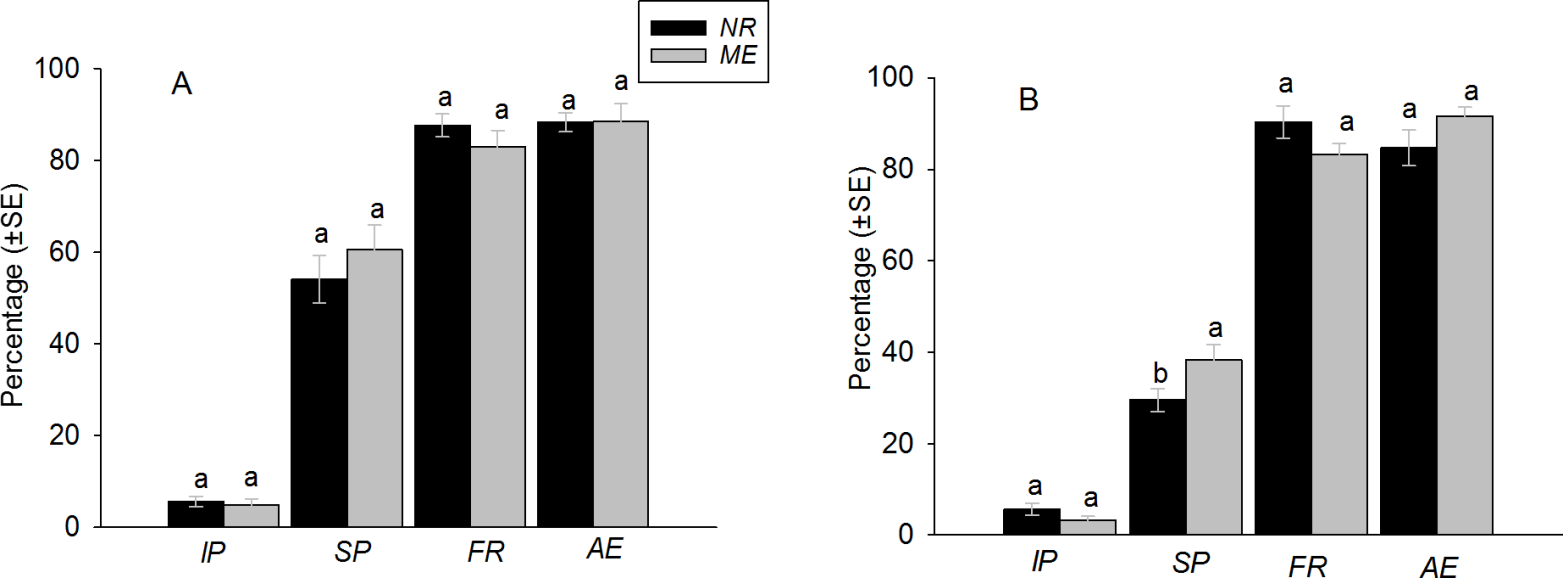
Percentage (mean ± SE) of incomplete parasitization (*IP*), successful parasitization (*SP*), female sex ratio (*FR*) or adult emergence (*AE*) of *Aphelinus abdominalis* when *Nasonovia ribisnigri* (*NR*) and *Macrosiphum euphorbiae* (*ME*) were offered as hosts under no-choice (A) or choice conditions (B) in laboratory experiments. Different letters above the bars indicate significant differences in *IP*, *SP*, *FR* or *AE* (Tukey’s or Mann-Whitney U-tests, p≤ 0.05).

This study showed that *A*. *abdominalis* has the ability to successfully parasitize two aphid species *N*. *ribisnigri* and *M*. *euphorbiae* when they were offered simultaneously or separately to a parasitoid on the same leaf discs. In no-choice situations, *A*. *abdominalis* successfully parasitized 54.02 ± 5.13% and 60.52 ± 5.35% of *N*. *ribisnigri* and *M*. *euphorbiae*, respectively, offered within a 24 h exposure period. There was no significant difference between in percent parasitism between the two aphid species (df = 1, 28; *F* = 0.94; *P* = 0.34) (Fig 1A). However, in the choice situation, there was a tendency for *A*. *abdominalis* successfully parasitizing more *M*. *euphorbiae* than *N*. *ribisnigri* (df = 1, 28; *F* = 4.04; *P =* 0.05), with parasitization of 38 ± 3.32% and 30 ± 2.50% respectively (Fig 1B). With respect to incomplete parasitization, a very low percentage (less than 6%) of aphids mortality occurred and no significant differences were detected when the two aphid species were offered simultaneously on the same leaf disc (df = 1,24; *F* = 0.01; *P* = 0.89) or on separate leaf discs (df = 1, 28; *F* = 0.00; *P* > 0.98) (Fig 1).

#### Adult emergence and sex ratio

More than 84% of mummified aphids emerged and a female-biased sex ratio (> 83%) was observed irrespective of aphid species (Fig 1). There was no significant difference in rates of parasitoid emergence and female sex ratio when the *N*. *ribisnigri* and the *M*. *euphorbiae* were exposed to *A*. *abdominalis* on separate leaf discs (parasitoid emergence: χ2 = 0.04; df = 1; P = 0.85; female sex ratio: χ2 = 1.02; df = 1; *P* = 0.311) or simultaneously (parasitoid emergence: χ2 = 1.71; df = 1; P = 0.19; female sex ratio: χ2 = 1.65; df = 1; *P* = 0.20) on the same leaf disc.

### Growth chamber experiments

**Fig 2:**
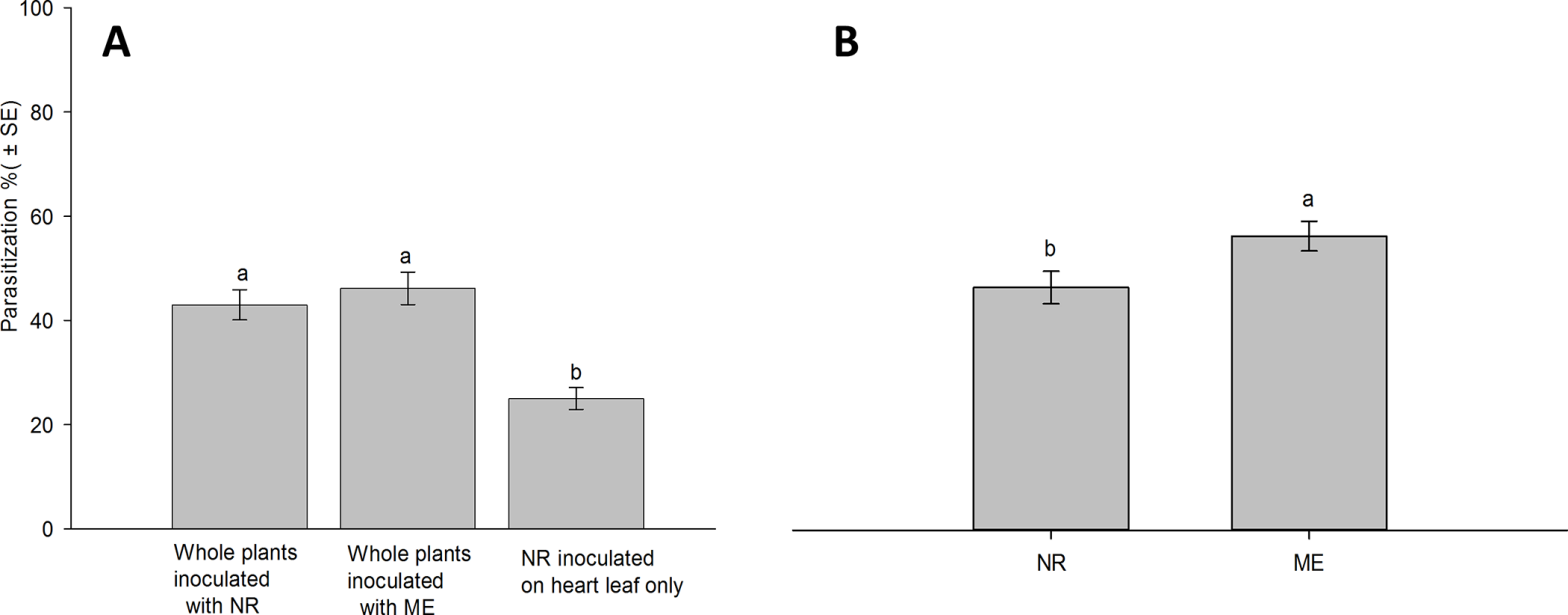
Percentage (mean ± SE) of successful parasitization of *Aphelinus abdominalis* when *Nasonovia ribisnigri* (*NR*) and *Macrosiphum euphorbiae* (*ME*) were offered as hosts under no- choice (A) or choice condition (B) in growth chamber experiments. Different letters above the bars indicate significant differences (Tukey’s test, p< 0.05).

This study showed that *A*. *abdominalis* has the capacity to find and parasitize not only aphids feeding on an exposed area (outer leaves) but also on a concealed area (lettuce heart leaf). There was a significant effect of aphid feeding location on the host finding ability of *A*. *abdominalis* in the no-choice tests (df = 2, 33; *F* = 17.46; *P* < 0.001). The percentage of *M*. *euphorbiae* or *N*. *ribisnigri* parasitized by *A*. *abdominalis* on whole lettuce plants was significantly higher than the percentage of *N*. *ribisnigri* parasitized when exposed only on the heart leaves (Fig 2A). There was no significant difference in the mummification of two aphid species exposed to parasitoids on whole plants.

In the choice test, however, when lettuce plants were inoculated with *M*. *euphorbiae* and *N*. *ribisnigri* simultaneously, a significant difference was detected in the degree of successful parasitization between two aphid species (df = 1, 22; *F* = 5.43; *P* = 0.03). *A*. *abdominalis* showed a significant preference to *M*. *euphorbiae* over *N*. *ribisnigri* (Fig 2B).

## Discussion

The understanding of parasitoid preference for parasitization between different aphid species, and the ability of parasitoid to find aphids feeding on different plant locations are important aspects in development of efficient biocontrol strategies against target pest populations [25, 26]. The choice experiment showed that *A*. *abdominalis* had a tendency for a higher successful parasitization in *M*. *euphorbiae* compared with *N*. *ribisnigri*, when they were offered simultaneously on the same leaf disc. Parasitoid preference for various aphid species have been examined previously [27, 28, 35, 36, 56]. For example, Bueno et al. [55] and Tepa-Yotto et al. [28] showed that *Lysiphlebus testaceipes* (Cresson) (Hymenoptera: Braconidae) preferred cotton aphid *Aphis gossypii* (Glover) as compared to three other aphid species: the green peach aphid, cowpea aphid *Aphis craccivora* (Koch) and mustard aphid *Lipaphis erysimi* (Kaltenbach). However, limited information exists regarding the parasitization preference of *A*. *abdominalis* between aphid species, except for the study by Wahab [57], who indicated that it preferred the shallot aphid *Myzus ascalonicus* (Doncaster) as compared with the ornate aphid *M*. *ornatus* (Laing) or mottled arum aphid *Neomyzus circumflexum* (Buckton).

Host quality [27, 28], parasitoid egg load [43], parasitoid age [42], aphid morph color [35, 36] and aphid size [27, 37] are important factors influencing parasitization preference of parasitoids between aphid species. Other factors may also mediate specialization and preferences of aphid parasitoids, including: 1) aphid phylogeny, with stronger preferences for closely related aphid species [30, 31]; 2) aphid host plant species, with greater preferences for aphids feeding on non-toxic host plants [31]; 3) the presence or absence of aphid endosymbiont bacteria, with higher preferences for non-infected aphids [33, 34].

The tendency to a higher preference of *A*. *abdominalis* for *M*. *euphorbiae* over *N*. *ribisnigri* found in our study is likely to be an effect of aphid size since the former species were relatively smaller in size (G. Shrestha, pers. obs.), and therefore presumably easier to handle, compared to the latter. Preference for small sized aphids was also observed for the parasitoid *Monoctonus paulensis* (Ashmead) (Hymenoptera: Braconidae) by Chau and Mackauer [27], who reported that small sized aphids have less well developed anti-parasitoid defense behaviour and therefore are easier to subdue. In addition, the green color of *M*. *euphorbiae* (as opposed to the red color of *N*. *ribisnigri*) could have played a role for the preference of *A*. *abdominalis* towards this aphid species [35, 36, 58]. For instance, Libbrecht et al. [36] reported that when parasitoids were given a choice, green morphs of pea aphid *Acyrthosiphon pisum* (Harris) were significantly more attacked by *Aphidius ervi* (Haliday) (Hymenoptera: Braconidae) when the neighboring colony consisted of red morphs.

The no-choice laboratory experiments showed no significant difference in degree of successful parasitization between the two aphid species; *N*. *ribisnigri* and *M*. *euphorbiae* and the parasitization percentages obtained for both aphid species are consistent with previous findings [22, 24]. This suggests that both aphid species are high quality hosts for *A*. *abdominalis* as it also substantiated by a high percentage of adult emergence and a strongly female-biased sex ratio being observed irrespective of aphid species. Sex ratio is important for aphid parasitoids, including *A*. *abdominalis*, as it affects parasitoid population growth rate and effectiveness in biocontrol [59, 60]. Parasitoids with female-biased sex ratios usually perform better in inoculative biocontrol programmes aimed at temporary establishment and reproduction in cropping systems [48, 59, 60]. Aphid parasitoid sex ratios can be influenced by a variety of host-related factors such as quality [31], age [48, 54], size [61] and species [44]. On suitable host aphid species, sex ratio in parasitoid offspring emerging from small hosts tend to be male-biased, and female-biased from intermediate or large hosts [29]. However, male-biased sex ratio in offspring from large hosts (fourth instar or adults) has been observed in some parasitoid species [54]. Our study found that sex ratios where female-biased on aphid nymphs of intermediate ages from both *N*. *ribisnigri* and *M*. *euphorbiae*, indicating that more female parasitoids emerged from higher quality hosts. This result supports the host quality model of Charnov and Skinner [62]. Similar results have also been reported for other aphid parasitoids [27, 44].

Irrespective of the different feeding locations of the aphids, the no-choice growth chamber experiments demonstrated that *A*. *abdominalis* has the capacity to find and parasitize aphids feeding both on outer leaves and on the heart leaves of lettuce. No reports are available on the effect of feeding locations on parasitization behavior of *A*. *abdominalis*. However, our results resemble the finding of Stadler and Volki [46] who reported that other parasitoids such as *Aphidius colemani* (Viereck) (Hymenoptera: Braconidae) partitioned parasitization or searching activity for banana aphids *Pentalonia nigronervosa* (Coquerel) between open and concealed areas of the banana plants. Our results, however, also showed that *A*. *abdominalis* parasitized a significantly lower proportion of aphids when offered *N*. *ribisnigri* inoculated only on heart leaves compared to when *N*. *ribisnigri* were offered on whole lettuce plants. This indicate that only a proportion of lettuce aphids located on the heart leaf were accessible to the parasitoid, presumably a result of some aphid being positioned on more open part and others on the deeper and more narrow part of the heart leaf (G. Shrestha, pers. obs.). In contrast with our findings, Stadler and Volki [46] showed that the parasitoid *L*. *testaceipes* parasitized *P*. *nigronervosa* only on open areas but not on the cryptic areas of banana plants. Thus, our and these previous illustrate that parasitoid ability to find hosts feeding on cryptic locations differ between parasitoid species, probably in combination with plant species morphology.

With respect to the choice growth chamber experiment, our study showed that *A*. *abdominalis* preferred to parasitize to *M*. *euphorbiae* as compared with *N*. *ribisnigri*. This will reduce the parasitoid’s ability to regulate populations of the *N*. *ribisnigri* when both aphid species occur simultaneously in lettuce plants as *A*. *abdominalis* will probably encounter and parasitize *M*. *euphorbiae* feeding on outer leaves [6] more frequently than *N*. *ribisnigri* feeding on heart leaves [7]. This is in accordance with results from a study by Gardner and Dixon [45], which showed that the rose grain aphid *Metopolophiurn dirhodum* (Walker) feeding on wheat leaves were parasitized more by *Aphidius rhopalosiphi* (DeStefani-Perez) (Hymenoptera: Braconidae) than the English grain aphid *Sitobion avenae* (Fabricius) feeding on the cryptic part (ear) of the wheat.

In conclusion, the present results indicate that *A*. *abdominalis* has a potential for inoculative biocontrol of *N*. *ribisnigri* and *M*. *euphorbiae*. The results suggest that the use of *A*. *abdominalis* against *N*. *ribisnigri* may not be adequate on its own, but that it may be considered as an additional option to be integrated with other potential biocontrol agents (e.g., predators and fungal entomopathogens) of *N*. *ribisnigri* [19, 21, 63]. Further long-term field or glasshouse studies that include several potential biocontrol agents and several aphid species are therefore needed in order to further validate the potential of biocontrol agents to suppress the *N*. *ribisnigri* population.
